# The Association Between Physical Activity and Uterine Leiomyoma and Its Symptoms: A Systematic Review and Meta‐Analysis

**DOI:** 10.1002/hsr2.70487

**Published:** 2025-02-23

**Authors:** Marcela Maria Birolim, Sara Carolina Scremin Souza, Renne Rodrigues, Danilo Fernandes da Silva, Vicente Martínez‐Vizcaíno, Arthur Eumann Mesas

**Affiliations:** ^1^ Department of Medicine Universidade Estadual do Centro Oeste Guarapuava Paraná Brazil; ^2^ Postgraduate Program in Health Promotion Centro Universitario Guairacá Guarapuava Paraná Brazil; ^3^ University of Ottawa Ottawa Canada; ^4^ Postgraduate Program in Public Health Universidade Estadual de Londrina Londrina Paraná Brazil; ^5^ Epidemiology Nucleus of Medical Graduation Universidade Federal da Fronteira Sul Chapecó Brazil; ^6^ Sports Studies Department, Faculty of Arts and Sciences Bishop's University Sherbrooke Quebec Canada; ^7^ Health and Social Research Center Universidad de Castilla‐La Mancha Cuenca Spain; ^8^ Facultad de Ciencias de la Salud Universidad Autónoma de Chile Talca Chile

**Keywords:** disease prevention, exercise, fibroids, women health

## Abstract

**Background and Aims:**

Although many studies have focused on the impact of physical activity on hormone‐mediated tumors, its effect on uterine leiomyomas (UL) remains unclear. This systematic review synthesizes the scientific evidence on the role of physical activity in the occurrence and symptomatology of UL while putting forward a research agenda.

**Methods:**

The PubMed, Scopus, Web of Science, SciELO, and Cochrane Library databases were searched for studies published up to January 10, 2025. The Newcastle‒Ottawa Scale was used to assess study quality, and the GRADE tool was used to determine evidence certainty. Dear‐Simonian and Laird random effects models were used to estimate the pooled odds ratio (OR) and 95% confidence interval (CI) of the association between physical activity and UL. The PRISMA guidelines were followed.

**Results:**

Fifteen studies were included (three cross‐sectional, 6 case–control, and 6 cohort studies), of which 11 were considered in the meta‐analysis. The difference between women who did and did not regularly practice physical activity (OR = 0.93; 95% CI: 0.82, 1.05; *I* ^2^ = 77.6%, *n* = 8 studies) in the likelihood of having UL did not meet conventional levels of statistical significance. Moreover, those women who engaged in more intense physical activity were less likely to have UL (OR = 0.86; 95% CI: 0.75, 0.99; *I* ^2^ = 80.5%, *n* = 5 studies) than those who engaged in less intense physical activity.

**Conclusion:**

Increased physical activity is associated with a slight decrease in the risk of UL and may provide relief from associated symptoms. Since current evidence is still limited to supporting specific physical activity recommendations, a research agenda is proposed for future studies on this subject.

**PROSPERO Registration:**

CRD42021247505.

## Summary

- **What Is Known**
  - While numerous studies have examined the impact of physical activity on hormone‐mediated tumors, its effect on uterine leiomyomas (UL) is still not well understood.
- **What Is New**
  - Regular physical activity is suggested to decrease the risk of developing UL and may help alleviate symptoms associated with UL.
  - Higher intensity of physical activity is associated with a lower risk of UL.
- **Clinical Implications**
  - Encouraging regular and higher‐intensity physical activity could be a beneficial strategy in reducing the risk and alleviating symptoms of UL.

## 1 Introduction

Uterine leiomyomas (UL), also known as fibroids, are among the most prevalent types of benign tumors in women [1, 2, 3], affecting approximately 6% of the global female population in 2019 [4] and generating substantial direct and indirect costs to individuals and the healthcare system [5]. These tumors can be asymptomatic or can result in chronic symptoms such as intense menstrual bleeding, pelvic pain, and dyspareunia [6, 7]. UL can be associated with infertility and pregnancy complications, which may impact the quality of life of women throughout the reproductive period [8, 9].

In addition to inherent aspects of genetics (e.g., ethnicity, family history) [10], hormonal variations across the female life cycle [2], and parity [11], some preventable and treatable clinical conditions, such as hypertension [2] and obesity [12], have been associated with UL. In an umbrella review of environmental risk factors related to UL, chronic physiological stress and obesity were graded as risk factors with suggestive evidence, while weak evidence was attributed to current alcohol intake as a risk factor, and oral contraceptive use and former smoking as protective factors [13]. Moreover, a recent systematic review on modifiable prognostic factors for the development of uterine fibroids identified evidence suggesting that maintaining adequate vitamin D levels, consuming more fruits and vegetables, and sustaining a normal body mass index (BMI) may serve as protective measures [14]. Regarding physical activity, this review [14] referenced a single study suggesting that exercise might be linked to a reduced risk of fibroid development among women of reproductive age, with the association appearing to depend on the intensity of exercise, even after accounting for BMI [15].

Exercise has been consistently associated with reduced incidence, growth, and metastasis of breast cancer [16], endometrial cancer [17], and other hormone‐related tumors, such as UL [18]. Similarly, physical activity may help reduce the likelihood of UL and alleviate symptoms. Evidence on the association between UL and physical activity is scarce, with limited and mixed results. While some studies support that regular physical activity is beneficial for the prevention of UL [15, 19, 20, 21], others have reported a lack of association [22, 23, 24]. The inconsistency between studies prevents the inclusion of generic recommendations for physical activity in UL management guidelines [25].

Therefore, the present study aimed to synthesize scientific evidence on the association between physical activity and UL. Furthermore, a research agenda is proposed to address the limitations of the existing evidence.

## 2 Methods

The present study is a systematic review and meta‐analysis conducted according to the Preferred Reporting Items for Systematic Review and Meta‐Analysis (PRISMA) recommendations, as described in the Supporting Information S1: Table S1. The review protocol was registered in PROSPERO (CRD42021247505).

The PubMed, Scopus, Web of Science, SciELO, and Cochrane Library databases were searched on January 10, 2025. For the search strategy, we used the Boolean operators “and” and “or” and the keywords and MeSH headings related to UL and physical activity. The detailed search strategy used for each database is presented in the Supporting Information S1: Table S2. The searches were limited to human studies, and no date or language restrictions were applied. The reference lists of all related systematic reviews found were revised to search for studies not captured in the main search.

The inclusion criteria were defined according to the PICO(S) strategy as follows: (i) Participants: adult women of reproductive age or in menopause or post menopause, (ii) Intervention or exposure: physical activity frequency or intensity, (iii) Comparison: physical inactivity or lower levels of physical activity, (iv) Outcome: UL or symptoms related to UL, and (v) Study design: peer‐reviewed observational and experimental studies. Studies involving nonuterine UL, review articles, studies with no data of interest on the association between physical activity and UL, and studies duplicating data from previous publications were excluded.

After excluding duplicate files, titles, and abstracts were screened to identify studies that potentially met the inclusion criteria. Then, a full‐text assessment of the remaining studies was carried out by two independent reviewers, and a third reviewer was consulted in case of discrepancies. The relevant data were extracted. In the study by Kim et al. [26], the data were extracted from one of the authors’ supplementary tables, summing the absolute numbers of women in their 20 s and 30 s who did or did not engage in regular physical activity and who did or did not have uterine fibroids.

The Newcastle‒Ottawa Scale (NOS) [27] was used to assess the quality of the included studies. The NOS assesses information and selection bias and confounding variables. Cohort and case–control studies can vary from 0 (lowest quality) to 9 (highest quality) stars based on the NOS. An adapted version of the NOS was used to evaluate cross‐sectional studies and varied from 0 to 8 stars (low to high quality). The questions and response answers of the adapted NOS for cross‐sectional studies can be found in the Supporting Information S1: Table S3 [28]. The study conducted by Huang et al. [29] although not classified as a cohort by the authors, was evaluated according to the quality criteria for cohort studies. The quality assessment for each included study was completed by two independent reviewers, and any discrepancies were resolved by consulting a third reviewer.

The Grades of Recommendations, Assessment, Development, and Evaluation (GRADE) [30] tool was used to determine the certainty of the evidence of the present systematic review.

The DerSimonian and Laird method was used to compute the pooled odds ratio (OR) and its respective 95% confidence interval (CI). The heterogeneity of the results across studies was assessed using the *I* ^2^ statistic. Meta‐analyses with random effects models were performed because moderate or high heterogeneity (*I* ^2^ > 50%) was observed. For this purpose, we first considered the frequency of physical activity as the exposure and UL as the outcome. In sequence, to assess a possible dose‒response relationship between physical activity intensity and UL, we grouped those studies with data on moderate versus low intensity and those with high versus low intensity.

Sensitivity analyses were conducted to assess the robustness of the summary estimates and to determine whether any study accounted for a large proportion of the heterogeneity. Meta‐regression and analysis of publication bias were not performed because these methods are not recommended when fewer than 10 studies are available for an outcome [31]. Statistical significance was set at a two‐sided *p* < 0.05. STATA SE 15 software (StataCorp, College Station, TX, USA) was used for the statistical procedures.

## 3 Results

**Figure 1:**
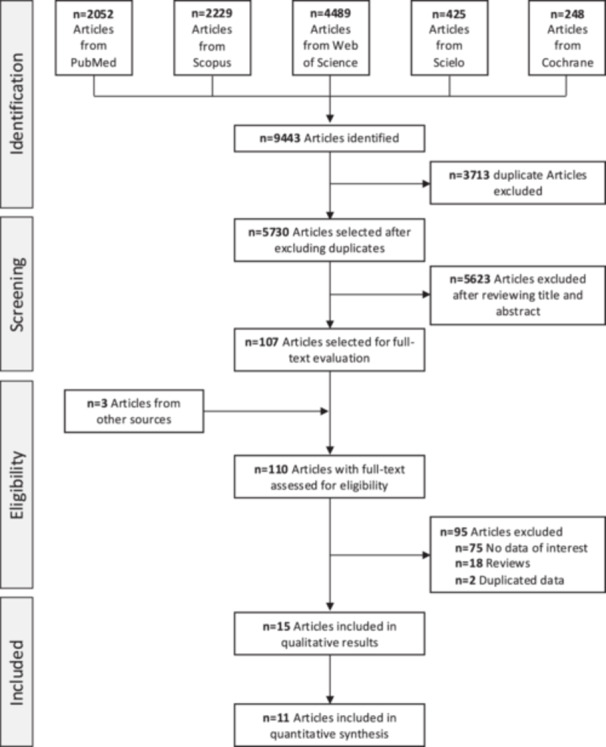
Flow diagram of the study selection process.

After deduplication, 5730 studies were identified for screening, 5623 of which were excluded after title and abstract review. Three studies from other sources (i.e., reference lists of related systematic reviews) were also identified and included for full‐text evaluation. Of the 110 articles selected for full‐text evaluation, 15 met the inclusion criteria and provided complete data on the study associations (Figure 1 and Supporting Information S1: Table S4). The included studies were published between 1986 and 2024, with sample sizes ranging from 83 to 2.4 million women. The characteristics of all included studies are summarized in Table 1.

**Table 1:** Characteristics of studies examining the association between uterine leiomyomas and physical activity.

First author, publication year	Design, sample size, age (years)	Population, country	Ethnicity/race	UL assessment	PA assessment (frequency or intensity, and categories)	Main results
Abdel Aziz and Mohamed, 2016 [19]	Case‒control, 300 women, 20–50 years	Gynecological outpatient clinic, with UL (cases, *n* = 150) and without UL (controls, *n* = 150), Egypt	Not reported	Diagnosed in a gynecological examination	IPAQ: low (< 600 MET‐min/week), moderate (600– < 1500 MET‐min/week) and high active (≥ 1500 MET‐min/week)	Higher PA levels were associated with a lower likelihood of having UL. High and moderate active women represented 10.7% and 33.3% of the case group, respectively, while the corresponding values for the control group were 30.0% and 50.0%.
Baird et al. 2007 [15]	Cross‐sectional, 1189 women, 35–49 years	Members of a health plan, USA	61.7% African American 38.3% Caucasian	Ultrasound; Surgical report; Self‐report	Self‐reported time of PA, without chores: Low (< 111 min/week); Medium (111–249 min/week); High (250–390 min/week); and Very high (> 390 min/week)	In adjusted analysis, a dose‒response pattern was observed in which women in the highest physical activity category were less likely to have UL. This trend was observed for African American (*p* = 0.070) and Caucasian women (*p* = 0.020).
Borah et al. 2013 [32]	Cross‐sectional, 968 women, 29–59 years	Women with a history of symptomatic UL, USA	64.2% Caucasian 28.1% African American 7.7% Other	Self‐reported diagnosis of symptomatic UL	Impact of UL in physical activities: All/most of the time, Little/some of time, None of time, Not applicable	The presence of UL impacts the performance of physical activities, 31.3% All/most of the time, 37.3% Little/some of time, 16.3% None of time, and 15.1% Not applicable, Young women (29–39 years old) were more affected than older women (*p* < 0.050).
Feofilova et al. 2018 [20]	Case‒control, 195 women, 18–62 years	Case (*n* = 98) of women hospitalized for surgical treatment of UL, and controls (*n* = 97) of regular female blood donor, Russian Federation	Not reported	Medical diagnosis in hospital admission	Self‐reported PA practice: daily, frequent (two to three times/week), infrequent (once a week) and extremely rare (once a month or less) versus lower frequency.	Women with daily or frequent (≥ two to three times/week) PA practice were less likely to be in the UL group than in the control group compared with those with infrequent or extremally rare PA frequency (*p* < 0.05).
He et al. 2013 [33]	Case‒control, 283 women, ≥ 18 years (maximum or average age not reported)	Women from a regular medical health checkup for gynecological diseases, with UL (cases, *n* = 73) and without UL (controls, n = 210), China	100% Asian	Ultrasound diagnosis or hysterectomies. The medical records of women who had undergone a hysterectomy were obtained.	IPAQ: Frequency: low (< 1 time/week), intermediate (1–2 time/week), and High (≥ 3 time/week); Intensity: low (walking or sedentary), intermediate (swimming or indoors sports), and high (running or ball game, etc.).	There was no significant difference regarding the PA frequency (high and intermediate of 50.9% and 21.1% for cases vs. 36.1% and 30.2% for controls, respectively) or intensity (high and intermediate 11.9% and 13.6% for cases vs. 9.9% and 11.7% for controls, respectively) (*p* > 0.05) and UL. Similar results were observed for PA time per week.
Huang et al. 2017 [29]	Retrospective cohort, 83 women, 21–35 years	Patients with UL who received ultrasound‐guided high‐intensity focused ultrasound (HIFU) treatment, China	100% Asian	UL (single intermural type with 3–6 cm) confirmed by enhanced MRI.	No exercise group (*n* = 51) versus Exercise group (*n* = 32), which received an “exercise intervention regimen” after HIFU with reinforcement.	Post‐HIFU exercise significantly improved UL resorption, reduced recurrence rate and increased the rate of pregnancy in UL patients
Jacoby et al. 2014 [34]	Prospective cohort, 933 women, 31–54 years	Premenopausal women with symptomatic UL, USA	40% Caucasian 31% African American 12% Latin American 11% Asian 5% Other	Self‐reported of diagnosed UL	Reported performing exercises to treat “pelvic problems” (yes or no).	40% of women with UL who did exercises reported that this “made symptoms a lot better,” while approximately 4% reported that exercise “bothered a lot or some by side effects.”
Kim et al. 2023 [26]	Retrospective cohort, 2,476,579 women, 20–39 years	Women subscriber of the Korean National Health Information Database, Republic of South Korea	100% Asian	Two outpatient records or one inpatient record of ICD‐10 codes of UL	Regular exercise (moderate physical activity for > 30 min/day and > 5 days/week or vigorous exercise > 3 days/week	Women reporting regular exercise were 7% more likely to present UL than those without physical activity (OR = 1.07; 95% CI: 1.05; 1.09).
Kim et al. 2024 [35]	Retrospective cohort, 672 women, 23–73 years	Participants who underwent health checkups at a hospital, the Republic of South Korea	Not reported	Ultrasound detection of ≥ 1 UL with ≥ 10 mm in length	Self‐reported PA quantified using MET‐min./week	No statistically significant difference in PA between women with (1037 MET‐min./week) or without (1140 MET‐min./week) UL (*p* > 0.99).
Muawad et al. 2022 [36]	Case‒control, 478 women, ≥ 18 (mean of 44.6 ± 8.83 years)	Women from obstetrics and gynecology clinics, with UL (cases, *n* = 239) and without UL (controls, *n* = 239), Kingdom of Saudi Arabia	Not reported	Ultrasound diagnosis and medical history	Self‐reported PA frequency: none, less than 3 h/week, 3–5 h/week, and ≥ 5 h/week	No statistically significant difference was found regarding PA frequency between cases (41.8%) and controls (46.0%) (*p* = 0.407).
Shen et al. 2013 [22]	Case‒control, 1,200 women, 30–50 years	Premenopausal women from gynecology and obstetrics service, with UL (cases, *n* = 600) and without UL (controls, *n* = 600), China	100% Asian	Ultrasound diagnosis and/or pathological examination for cases, healthy volunteers for controls	Self‐reported PA frequency: frequent, occasional, and never.	No statistically significant difference regarding PA frequency was observed between cases (2.0% frequent, 18.3% occasional) and controls (2.7% frequent, 17.3% occasional).
Tak et al. 2016 [24]	Case‒control, 1,230 women, ≥ 18 years (mean of 44 years, interquartile range: 40–47)	Premenopausal women checked for UL in a hospital, with UL (cases, n = 615) and without UL (controls, *n* = 615), Republic of South Korea	Not reported	Transvaginal ultrasonography with histological confirmation for cases, Transvaginal ultrasonography and medical history for controls.	Self‐reported PA frequency: regular (≥ 3 times/week), and not regular (≤ 2 times/week).	No statistically significant difference regarding regular PA frequency was observed between cases (26%) and controls (31%) (*p* = 0.099).
Uimari et al. 2016 [23]	Prospective cohort, 3635 women, 46 years	Women from a birth cohort, Finland	100% Caucasian	Medical history in healthcare system or self‐reported diagnosed UL confirmed by ultrasonography or surgery.	Self‐reported PA frequency: inactive, lightly active, active or very active.	No statistically significant difference regarding PA frequency was observed between cases (43.7% lightly, 34.9% active, and 1.3% very active) and controls (41.0% lightly, 35.5% active, and 1.6% very active) (*p* = 0.50).
Wise et al. 2016 [37]	Prospective cohort, 47,267 women, 21–69 years	Women who subscribe *Essence* magazine and black professional organizations (Black Women's Health Study), USA	100% African American	Self‐reported diagnosed UL	Self‐reported of PA time: none (0 h/week), moderate (1–4 h/week), vigorous (≥ 5 h/week)	No statistically significant difference regarding vigorous PA was observed between cases (13.5%) and controls (13.8%) at baseline.
Wyshak et al. 1986 [21]	Cross‐sectional, 5398 women, 21–80 years	Former college‐athletes/nonathletes, USA	Not reported	Self‐reported history of benign tumors of the uterus	A woman was considered an athlete when she was a member of at least one varsity, house, or another intramural team for at least 1 year and/or earned another sports award.	Nonathlete women had a higher age‐adjusted risk ratio for benign uterine tumors than athlete women (RR = 1.40; 95% CI: 1.03–1.89). Athletes presented age‐adjusted rates of 30.6/1,000 benign uterine tumors versus 42.6/1000 benign uterine tumors of nonathletes.

Abbreviations: CI, confidence interval; IPAQ, International Physical Activity Questionnaire; MET, metabolic equivalent; OR, odds ratio; PA, physical activity; RR, relative risk; UL, uterine leiomyoma.

With regard to the design of the included studies, six were case–control studies [19, 20, 22, 24, 33, 36], six were cohort studies [23, 26, 29, 34, 35, 38], and three were cross‐sectional [15, 21, 32]. No experimental study met the inclusion criteria. Among the three prospective cohort studies, the mean follow‐up time ranged from 4.2 (annual follow‐up) [34] to 46.0 years (follow‐up conducted at years 1, 14, 31, and 46) [23].

Among the 11 studies reporting data on the association between physical activity and UL, four found favorable associations between practice or greater frequency and/or intensity of physical activity and a lower frequency of UL [15, 19, 20, 21], six did not find such an association [22, 23, 24, 33, 35, 36], and one reported an inverse association, with a greater risk of UL among women practicing regular physical activity compared with those without this behavior [26]. In the study by Jacoby et al. [34] in which all women had UL, physical activity was associated with an improvement in UL‐related symptoms. In another study, Huang et al. [29] reported that exercise after high‐intensity focused ultrasound treatment significantly improved uterine fibroid absorption, decreased the recurrence rate, and enhanced the pregnancy rate in uterine fibroid patients [29]. Moreover, the authors found that exercise led to a more significant reduction in the dysmenorrhea score [29]. Borah et al. [32] investigated the inverted association (i.e., the impact of UL on physical activity) and suggested that the presence of UL can lead to a reduction in physical activity levels throughout the lifespan. Wise et al. [37] used physical activity practice only as a covariate for the analyses, and no significant differences were found between black women with (13.5%) and without UL (13.8%) at baseline.

The quality of the included studies ranged between 5 and 8 stars for cross‐sectional studies and between 4 and 8 stars for cohort and case–control studies (Supporting Information S1: Table S5). The GRADE assessment revealed an overall low quality of evidence, mainly attributed to imprecision of the results and the potential risk of bias. The full details are provided in Supporting Information S1: Table S6.

**Figure 2:**
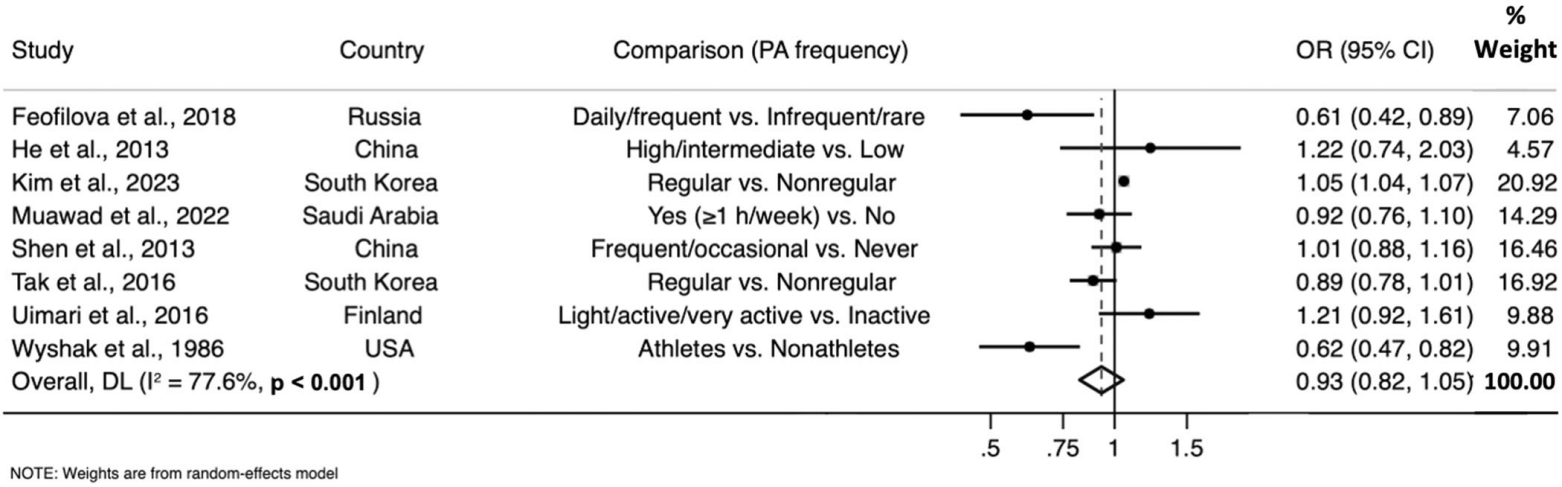
Forest plot of the association between the frequency of physical activity and uterine leiomyomas (CI, confidence interval; OR, odds ratio).

Figure 2 depicts the forest plot of studies on the frequency of physical activity and UL. The pooled OR revealed that the difference between women who did and did not regularly practice physical activity (OR = 0.93; 95% CI: 0.82, 1.05; I2 = 77.6%, *n* = 8 studies) in the likelihood of having UL did not meet conventional levels of statistical significance. Two studies showed significant beneficial effects of physical activity on UL [20, 21]. Feofilova et al. reported that women with daily or frequent (≥ 2–3 times/week) outdoor physical activity practices were less likely to be in the UL group than in the control group [20]. In the study by Wyshak et al. [21] nonathlete women had a greater age‐adjusted risk ratio for benign uterine tumors when compared with athlete women. Moreover, the association between the frequency of physical activity and UL was not observed in the sensitivity analyses when each of the included studies was removed (Supporting Information S1: Table S7).

**Figure 3:**
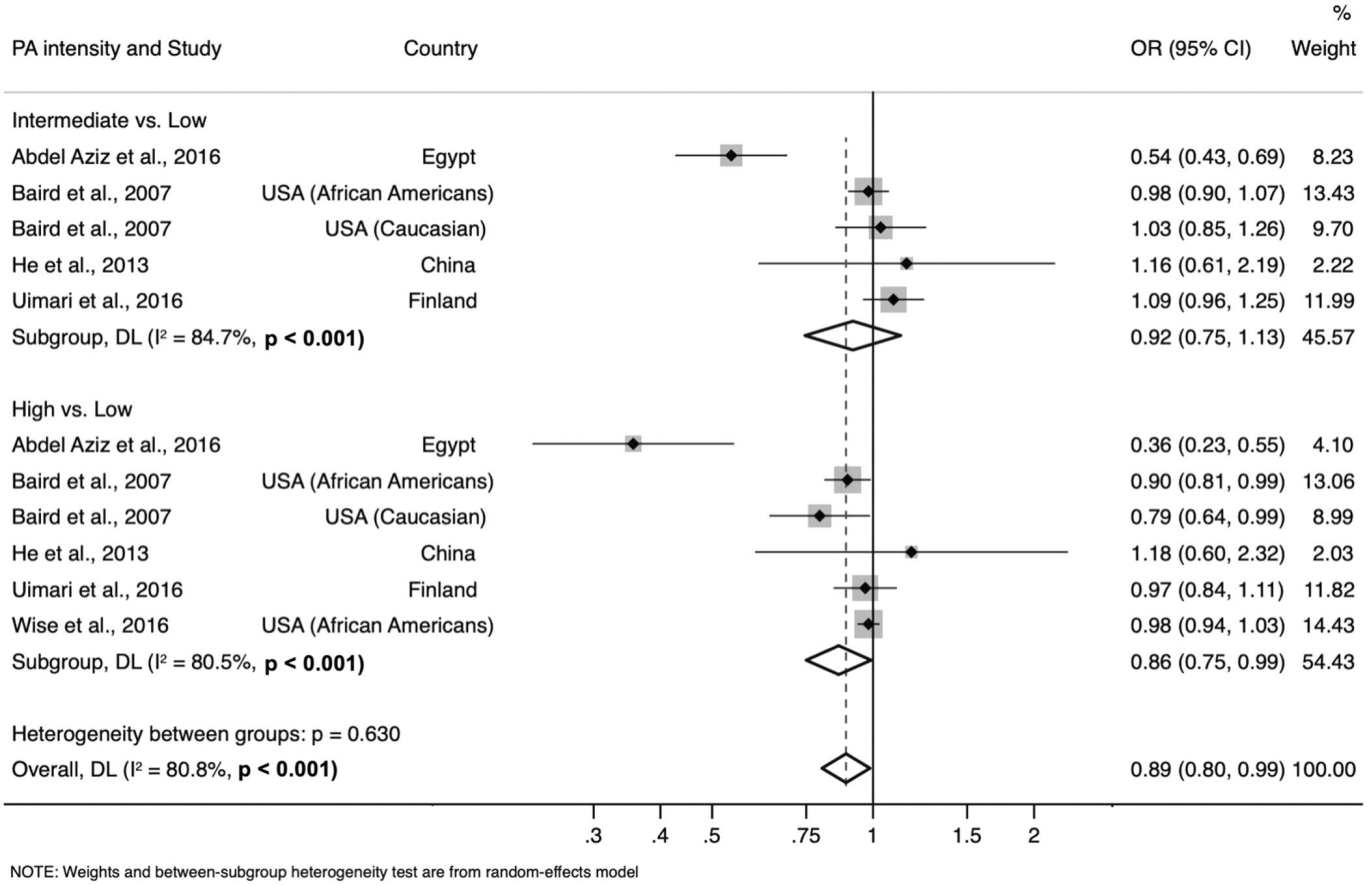
Forest plot of the association between physical activity intensity and uterine leiomyomas. (CI, confidence interval; OR, odds ratio).

As presented in Figure 3, no association was detected between moderate‐ versus low‐intensity physical activity and the occurrence of UL (OR = 0.92; 95% CI: 0.75, 1.13; *I* ^2^ = 84.7%, *n* = 4 studies). However, women who practiced physical activity more intensively were less likely to have UL (OR = 0.86; 95% CI: 0.75, 0.99; *I* ^2^ = 80.5%, *n* = 5 studies) than those who practiced low‐intensity physical activity. Overall, UL was less likely to occur when comparing high‐ or moderate‐intensity physical activity with low‐intensity physical activity (OR = 0.89; 95% CI: 0.80, 0.99; *I* ^2^ = 80.8%, *n* = 5 studies). In the sensitivity analysis, the main result remained significant except when the data from Baird et al. [15] involving African American women, and the study of Abdel Aziz et al. [19] were excluded (Supporting Information S1: Table S8).

## 4 Discussion

This study summarizes the available evidence on the association between physical activity and UL. Overall, this systematic review showed a similar distribution between studies that reported favorable results for the potential benefits of physical activity on UL and studies that reported no association. In the meta‐analysis, the frequency of physical activity was not associated with UL, whereas a higher intensity of physical activity was linked to a reduced likelihood of UL. However, it is noteworthy that this association was not observed when two studies [15, 19] were excluded from the sensitivity analysis. In addition, other studies have provided preliminary evidence that physical activity can act as an adjuvant in the treatment of UL [29] and improve UL‐related symptomatology [34]. Finally, it has been reported that the presence of UL can decrease physical activity levels [32]. Although most of the results are favorable for physical activity as a strategy to improve UL occurrence and conditions, the quality of the studies currently available is limited, and the level of evidence is not sufficient to support individual and population‐based recommendations.

Although the most recent physical activity guidelines [39] highly recommend exercise to improve metabolic health, physical activity frequency is less informative and understudied than other physical activity principles, such as the type, intensity, and volume of physical activity. In our results, two studies revealed an association between frequent physical activity and a decreased likelihood of reporting UL. One study compared high (≥ 3 times/week) versus low (≤ 1 time/week) frequencies of physical activity [20], while the other study compared athletes (i.e., more frequently exercisers) versus nonathletes [21]. It is to be expected that women who practice exercise more frequently (such as athletes) are also more exposed to higher exercise intensities, which allows them to enhance their physical and mental health performance. The study with the largest sample size (almost 2.5 million Korean women) provided a conflicting result compared with others and the initial hypothesis, revealing, through a retrospective cohort design, a small yet significant increase in the risk of fibroids in women who engaged in regular physical activity compared with those who did not [26]. This finding was not further explored in the study because its focus was on alcohol consumption and UL [26]. It could be speculated that this result is due to the eventual type 1 error observed in epidemiological studies with large samples; however, it cannot be ruled out that the result is due to uncontrolled confounding factors in the analyses, such as age, BMI, or diet.

The results on the association between physical activity intensity and UL are similar to those observed when considering the frequency component. No difference was found between low‐ and moderate‐intensity physical activity in terms of the likelihood of reporting UL, but an association was observed when comparing high‐ to low‐intensity physical activity, favoring the greater intensity group. Considering the small number of included studies and the substantial unexplained heterogeneity between them, it was not possible to determine whether there was a dose–response effect in the meta‐analysis. One of the included studies reported a trend toward a reduction in the incidence of UL due to increased physical activity intensity [15]. Although the dose–response effect is supported by that study [15], this effect should be investigated in prospective studies focused on exploring different levels of physical activity intensity and its effects on UL.

Only one study found in this systematic review showed that physical activity can be an adjuvant to standard therapies to reduce the volume and number of UL [29]. In addition, other findings suggested that symptoms related to UL improved among women who practiced physical activity [34]. The typical symptoms of uterine fibroids, such as heavy menstrual bleeding, clotting, spotting between periods, gastrointestinal changes, and pelvic pressure, can place a heavy burden on a woman's life [40]. Both reduced UL size and decreased symptoms, likely associated with physical activity, can significantly improve the quality of life of women [32]. Therefore, the adequate prescription and management of physical activity could be an alternative to potentialize the results of traditional treatments for UL, such as the use of medication or surgery [41, 42].

Several mechanisms have been proposed to explain why physical activity can be beneficial for preventing and alleviating UL‐related symptoms. Physical activity is known to be a protective factor against other types of estrogen‐dependent tumors, such as breast [43] and endometrial tumors [44], so its potential positive effects on UL may occur through similar pathways. Some pathways related to the modulation of hormones and biomarkers linked to adipose tissue have been proposed to explain the reduction in hormone‐dependent tumors in response to physical activity [45]. First, decreased fat mass may inhibit the conversion of adrenal androgens to estrogens that occur in adipose tissue, leading to a decreased stimulus for tumor cell proliferation [45, 46]. Second, the overall decrease in body weight can reduce cholesterol biosynthesis, which decreases estrogen and progesterone production and consequently tumor induction [47]. Third, decreased secretion of adipokines and inflammatory cytokines may improve insulin resistance [48] and the consequent development of UL [46]. Modulation of the intestinal microbiome [49] and epigenetic mechanisms [3, 46, 49] resulting from the synergistic effect between physical activity and other healthy lifestyle habits, especially diet [46, 50], may also be involved in the physical activity‐UL relationship.

The present review has limitations that should be considered when interpreting the findings. First, the number of studies included in the meta‐analysis was insufficient to analyze heterogeneity not explained by subgroups or meta‐regression. Second, methodological differences, such as the type of study design and lack of representativeness of the samples of the included studies, limit the generalizability of the results. Third, four studies were based on self‐reports of diagnosed UL [21, 32, 34, 37], which may have led to information bias. A similar potential bias may apply to the methods used to assess physical activity, which were mostly based on self‐reports. Finally, except for the study by Baird et al. [15] which adjusted their analysis for important confounders, all other studies presented only unadjusted analyses. Thus, it cannot be ruled out that the benefits attributed to physical activity are due, at least in part, to other variables not considered, such as age, family history of UL, use of drugs, hormone therapy, and dietary pattern.

Considering the limitations mentioned above, we propose recommendations to enhance the quality and broaden the applicability of future research on the effects of physical activity on UL. First and foremost, it is essential to conduct targeted randomized controlled trials (RCTs) to evaluate the potential benefits of physical activity for women with symptomatic UL. For example, even the use of minimally invasive myomectomy is controversial due to concerns about surgical complexity, safety, long‐term outcomes, and the potential risk of inadvertently treating an undiagnosed sarcoma [51, 52]. RCTs are therefore needed to assess the cost‐effectiveness of nonpharmacological strategies to prevent these tumors and relieve associated symptoms (e.g., regular exercise, physical activity promotion) [14, 53]. Additionally, prospective observational studies (cohorts) with long‐term follow‐up spanning reproductive and postmenopausal stages should be conducted to establish causal relationships between physical activity and a reduced risk of UL. Given the multifactorial nature of UL and the influence of sociocultural factors, it is particularly important to include women from diverse ethnic backgrounds, including those from countries in the Southern Hemisphere, such as Latin America and Africa. When designing both clinical and population studies, it is crucial to capture the frequency, intensity, and type of physical activity to formulate personalized recommendations. Moreover, future studies should provide comprehensive information on outcome variables such as the occurrence, location, number, and size of UL or associated symptoms. Finally, thorough consideration should be given to collecting data on potential confounding factors, as mentioned earlier.

In conclusion, the results of this systematic review and meta‐analysis suggest that women who engage in high‐intensity physical activity may be less likely to have UL than those who engage in low‐intensity or no physical activity. However, in addition to the fact that most studies used self‐reported physical activity, which is subject to information bias, the available evidence is insufficient in terms of quantity and quality to make recommendations about physical activity practices and the prevention or treatment of UL. Furthermore, the significant heterogeneity among the studies prevents direct comparisons between them, and caution is needed when interpreting the combined results of the meta‐analysis.

## Author Contributions

**Marcela Maria Birolim:** conceptualization, methodology, investigation, writing–original draft, writing–review and editing. **Sara Carolina Scremin Souza:** conceptualization, methodology, investigation, writing–original draft, writing–review and editing. **Renne Rodrigues:** conceptualization, methodology, investigation, writing–original draft, writing–review and editing. **Danilo Fernandes da Silva:** conceptualization, methodology, investigation, writing– original draft, writing – review and editing. **Vicente Martínez‐Vizcaíno:** conceptualization, formal analysis, writing–review and editing. **Arthur Eumann Mesas:** conceptualization, methodology, formal analysis, investigation, data curation, writing–original draft, writing–review and editing, supervision.

## Conflicts of Interest

The authors declare no conflicts of interest.

5

### Data Availability Statement

The authors confirm that the data supporting the findings of this systematic review are available within the studies included or its supplementary materials.

## Transparency Statement

The lead author Arthur Eumann Mesas affirms that this manuscript is an honest, accurate, and transparent account of the study being reported; that no important aspects of the study have been omitted; and that any discrepancies from the study as planned (and, if relevant, registered) have been explained.

## Supporting information

Supporting information.

## References

[1] D. Day Baird , D. B. Dunson , M. C. Hill , D. Cousins , and J. M. Schectman , “High Cumulative Incidence of Uterine Leiomyoma in Black and White Women: Ultrasound Evidence,” American Journal of Obstetrics and Gynecology 188 (2003): 100–107. 12548202 10.1067/mob.2003.99

[2] E. Stewart , C. Cookson , R. Gandolfo , and R. Schulze‐Rath , “Epidemiology of Uterine Fibroids: A Systematic Review,” BJOG: An International Journal of Obstetrics & Gynaecology 124 (2017): 1501–1512. 28296146 10.1111/1471-0528.14640

[3] Q. Yang , M. Ciebiera , M. V. Bariani , et al., “Comprehensive Review of Uterine Fibroids: Developmental Origin, Pathogenesis,” Endocrine Reviews 43 (2022): 678–719. 34741454 10.1210/endrev/bnab039 PMC9277653

[4] “Global Burden of Disease. The Global Burden of Cancer Attributable to Risk Factors, 2010–19: A Systematic Analysis for the Global Burden of Disease Study 2019,” Lancet 400 (2022): 563–591. 35988567 10.1016/S0140-6736(22)01438-6 PMC9395583

[5] A. M. Soliman , H. Yang , E. X. Du , S. S. Kelkar , and C. Winkel , “The Direct and Indirect Costs of Uterine Fibroid Tumors: A Systematic Review of the Literature Between 2000 and 2013,” American Journal of Obstetrics and Gynecology 213 (2015): 141–160. 25771213 10.1016/j.ajog.2015.03.019

[6] K. H. Kjerulff , P. Langenberg , J. D. Seidman , P. D. Stolley , and G. M. Guzinski , “Uterine Leiomyomas. Racial Differences in Severity, Symptoms and Age at Diagnosis,” Journal of Reproductive Medicine 41 (1996): 483–490. 8829060

[7] G. Wegienka , G. Wegienka , D. D. Baird , et al., “Self‐Reported Heavy Bleeding Associated With Uterine Leiomyomata,” Obstetrics & Gynecology 101 (2003): 431–437. 12636944 10.1016/s0029-7844(02)03121-6

[8] L. I. Zepiridis , G. F. Grimbizis , and B. C. Tarlatzis , “Infertility and Uterine Fibroids,” Best Practice & Research Clinical Obstetrics & Gynaecology 34 (2016): 66–73. 26856931 10.1016/j.bpobgyn.2015.12.001

[9] L. M. Coutinho , W. A. Assis , A. Spagnuolo‐Souza , and F. M. Reis , “Uterine Fibroids and Pregnancy: How Do They Affect Each Other?,” Reproductive Sciences 29 (2022): 2145–2151. 34142343 10.1007/s43032-021-00656-6

[10] L. A. Wise , J. R. Palmer , E. A. Stewart , and L. Rosenberg , “Age‐Specific Incidence Rates for Self‐Reported Uterine Leiomyomata in the Black Women's Health Study,” Obstetrics & Gynecology 105 (2005): 563–568. 15738025 10.1097/01.AOG.0000154161.03418.e3 PMC1847590

[11] D. Day Baird and D. B. Dunson , “Why Is Parity Protective for Uterine Fibroids?,” Epidemiology 14 (2003): 247–250. 12606893 10.1097/01.EDE.0000054360.61254.27

[12] H. Qin , Z. Lin , E. Vásquez , X. Luan , F. Guo , and L. Xu , “Association between Obesity and the Risk of Uterine Fibroids: A Systematic Review and Meta‐Analysis,” Journal of Epidemiology and Community Health 75 (2021): 197–204. 33067250 10.1136/jech-2019-213364

[13] A. M. Salehi , E. Jenabi , S. Farashi , S. Aghababaei , and Z. Salimi , “The Environmental Risk Factors Related to Uterine Leiomyoma: An Umbrella Review,” Journal of Gynecology Obstetrics and Human Reproduction 52 (2023): 102517. 36481492 10.1016/j.jogoh.2022.102517

[14] A. L. Keizer , A. Semmler , H. S. Kok , P. J. M. van Kesteren , J. A. F. Huirne , and W. J. K. Hehenkamp , “Modifiable Prognostic Factors in Uterine Fibroid Development: A Systematic Review of Literature,” Journal of Obstetrics and Gynaecology 44 (2024): 2288225. 38102975 10.1080/01443615.2023.2288225

[15] D. D. Baird , D. B. Dunson , M. C. Hill , D. Cousins , and J. M. Schectman , “Association of Physical Activity With Development of Uterine Leiomyoma,” American Journal of Epidemiology 165 (2007): 157–163. 17090618 10.1093/aje/kwj363

[16] A. McTiernan , C. M. Friedenreich , P. T. Katzmarzyk , et al., “Physical Activity in Cancer Prevention and Survival: A Systematic Review,” Medicine & Science in Sports & Exercise 51 (2019): 1252–1261. 31095082 10.1249/MSS.0000000000001937 PMC6527123

[17] A. E. Cust , B. K. Armstrong , C. M. Friedenreich , N. Slimani , and A. Bauman , “Physical Activity and Endometrial Cancer Risk: A Review of the Current Evidence, Biologic Mechanisms and the Quality of Physical Activity Assessment Methods,” Cancer Causes & Control 18 (2007): 243–258. 17206535 10.1007/s10552-006-0094-7

[18] R. Sparic , L. Mirkovic , A. Malvasi , and A. Tinelli , “Epidemiology of Uterine Myomas: A Review,” International Journal of Fertility & Sterility 9 (2016): 424–435. 26985330 10.22074/ijfs.2015.4599 PMC4793163

[19] N. Abdel Aziz and S. Mohamed , “Relationship between Diet, Physical Activity and the Development of Uterine Fibroids,” Alexandria Scientific Nursing Journal 18 (2016): 15–32.

[20] M. A. Feofilova , O. G. Pavlov , and V. E. Geimerling , “The Effect of Life‐Style and Occupational Hazards on Development of Hysteromyoma,” Problemy Sotsial'noi Gigieny, Zdravookhraneniia I Istorii Meditsiny 26 (2018): 406–410. 10.32687/0869-866X-2018-26-6-406-410 30748132

[21] G. Wyshak , R. E. Frisch , N. L. Albright , T. E. Albright , and I. Schiff , “Lower Prevalence of Benign Diseases of the Breast and Benign Tumours of the Reproductive System Among Former College Athletes Compared to Non‐Athletes,” British Journal of Cancer 54 (1986): 841–845. 3801278 10.1038/bjc.1986.249 PMC2001555

[22] Y. Shen , Q. Xu , J. Xu , M. L. Ren , and Y. L. Cai , “Environmental Exposure and Risk of Uterine Leiomyoma: An Epidemiologic Survey,” European Review for Medical and Pharmacological Sciences 17 (2013): 3249–3256. 24338469

[23] O. Uimari , J. Auvinen , J. Jokelainen , et al., “Uterine Fibroids and Cardiovascular Risk,” Human Reproduction 31 (2016): 2689–2703. 27733532 10.1093/humrep/dew249

[24] Y. J. Tak , S. Y. Lee , S. K. Park , et al., “Association Between Uterine Leiomyoma and Metabolic Syndrome in Parous Premenopausal Women: A Case‐Control Study,” Medicine 95 (2016): e5325. 27861360 10.1097/MD.0000000000005325 PMC5120917

[25] G. A. Vilos , C. Allaire , P. Y. Laberge , et al., “The Management of Uterine Leiomyomas,” Journal of Obstetrics and Gynaecology Canada 37 (2015): 157–178. 25767949 10.1016/S1701-2163(15)30338-8

[26] S. Kim , K. Han , S. Y. Choi , et al., “Alcohol Consumption and the Risk of New‐Onset Uterine Leiomyomas: A Nationwide Population‐Based Study in 2.5 Million Korean Women Aged 20 to 39 Years,” American Journal of Obstetrics and Gynecology 229 (2023): 45.e1–45.e18. 10.1016/j.ajog.2023.03.041 37023913

[27] G. Wells , B. Shea , D. O'Connell , et al., “The Newcastle–Ottawa Scale (NOS) for Assessing the Quality of Nonrandomised Studies in Meta‐Analyses. Secondary The Newcastle–Ottawa Scale (NOS) for Assessing the Quality of Nonrandomised Studies in Meta‐Analyses,” Ottawa Hospital Research Institute 18 (2015): 2–4.

[28] A. Moskalewicz and M. Oremus , “No Clear Choice Between Newcastle‐Ottawa Scale and Appraisal Tool for Cross‐Sectional Studies to Assess Methodological Quality in Cross‐Sectional Studies of Health‐Related Quality of Life and Breast Cancer,” Journal of Clinical Epidemiology 120 (2020): 94–103. 31866469 10.1016/j.jclinepi.2019.12.013

[29] X. Huang , D. Yu , M. Zou , L. Wang , H. Xing , and Z. Wang , “The Effect of Exercise on High‐Intensity Focused Ultrasound Treatment Efficacy in Uterine Fibroids and Adenomyosis: A Retrospective Study,” BJOG: An International Journal of Obstetrics & Gynaecology 124, no. Suppl (2017): 46–52. 28856860 10.1111/1471-0528.14748

[30] G. H. Guyatt , A. D. Oxman , G. E. Vist , et al., “Grade: an Emerging Consensus on Rating Quality of Evidence and Strength of Recommendations,” BMJ 336 (2008): 924–926. 18436948 10.1136/bmj.39489.470347.AD PMC2335261

[31] J. J. Deeks , J. P. T. Higgins and D. G. Altman , ed., “Chapter 10: Analysing Data and Undertaking Meta‐Analyses.” in Cochrane Handbook for Systematic Reviews of Interventions version 6.4, ed. J. P. T. Higgins , J. Thomas , J. Chandler , M. Cumpston , T. Li , M. J. Page , and V. A. Welch (Cochrane, 2023), www.training.cochrane.org/handbook.

[32] B. J. Borah , W. K. Nicholson , L. Bradley , and E. A. Stewart , “The Impact of Uterine Leiomyomas: A National Survey of Affected Women,” American Journal of Obstetrics and Gynecology 209 (2013): 319.e1–319.e20. 10.1016/j.ajog.2013.07.017 PMC4167669 23891629

[33] Y. He , Q. Zeng , S. Dong , L. Qin , G. Li , and P. Wang , “Associations Between Uterine Fibroids and Lifestyles Including Diet, Physical Activity and Stress: A Case‐Control Study in China,” Asia Pacific Journal of Clinical Nutrition 22 (2013): 109–117. 23353618 10.6133/apjcn.2013.22.1.07

[34] V. L. Jacoby , A. Jacoby , L. A. Learman , et al., “Use of Medical, Surgical and Complementary Treatments Among Women With Fibroids,” European Journal of Obstetrics & Gynecology and Reproductive Biology 182 (2014): 220–225. 25445104 10.1016/j.ejogrb.2014.09.004 PMC4630000

[35] M. J. Kim , S. Kim , J. J. Kim , et al., “Dietary Intake Is Associated With the Prevalence of Uterine Leiomyoma in Korean Women: A Retrospective Cohort Study,” PLoS One 19, no. 2 (2024): e0291157. 38359002 10.1371/journal.pone.0291157 PMC10868850

[36] R. Muawad , R. Dabbagh , and Y. Sabr , “Association of Health and Lifestyle Factors With Uterine Fibroids Among Saudi Women: A Case‐Control Study,” Journal of Taibah University Medical Sciences 17 (2022): 1039–1046. 36212583 10.1016/j.jtumed.2022.06.005 PMC9519786

[37] L. A. Wise , T. R. Sponholtz , L. Rosenberg , et al., “History of Uterine Leiomyoma and Risk of Endometrial Cancer in Black Women,” Cancer Causes & Control 27 (2016): 545–552. 26923705 10.1007/s10552-016-0728-3 PMC4798859

[38] L. A. Wise , R. G. Radin , L. Rosenberg , L. Adams‐Campbell , and J. R. Palmer , “History of Uterine Leiomyomata and Incidence of Breast Cancer,” Cancer Causes & Control 26 (2015): 1487–1493. 26250515 10.1007/s10552-015-0647-8 PMC4567934

[39] F. C. Bull , S. S. Al‐Ansari , S. Biddle , et al., “World Health Organization 2020 Guidelines on Physical Activity and Sedentary Behaviour,” British Journal of Sports Medicine 54 (2020): 1451–1462. 33239350 10.1136/bjsports-2020-102955 PMC7719906

[40] M. Fuldeore and A. Soliman , “Patient‐Reported Prevalence and Symptomatic Burden of Uterine Fibroids Among Women in the United States: Findings From a Cross‐Sectional Survey Analysis,” International Journal of Women's Health 9 (2017): 403–411. 10.2147/IJWH.S133212 PMC5476627 28652819

[41] J. Donnez and M. M. Dolmans , “Uterine Fibroid Management: From the Present to the Future,” Human Reproduction Update 22 (2016): 665–686. 27466209 10.1093/humupd/dmw023 PMC5853598

[42] M. S. De La Cruz and E. M. Buchanan , “Uterine Fibroids: Diagnosis and Treatment,” American Family Physician 95 (2017): 100–107. 28084714

[43] M. C. de Boer , E. A. Wörner , D. Verlaan , and P. A. M. van Leeuwen , “The Mechanisms and Effects of Physical Activity on Breast Cancer,” Clinical Breast Cancer 17 (2017): 272–278. 28233686 10.1016/j.clbc.2017.01.006

[44] O. Raglan , I. Kalliala , G. Markozannes , et al., “Risk Factors for Endometrial Cancer: An Umbrella Review of the Literature,” International Journal of Cancer 145 (2019): 1719–1730. 30387875 10.1002/ijc.31961

[45] B. S. Hong and K. P. Lee , “A Systematic Review of the Biological Mechanisms Linking Physical Activity and Breast Cancer,” Physical Activity and Nutrition 24 (2020): 25–31. 33108715 10.20463/pan.2020.0018 PMC7669467

[46] A. AlAshqar , L. Reschke , G. W. Kirschen , and M. A. Borahay , “Role of Inflammation in Benign Gynecologic Disorders: From Pathogenesis to Novel Therapies,” Biology of Reproduction 105 (2021): 7–31. 33739368 10.1093/biolre/ioab054 PMC8256101

[47] L. Crudele , E. Piccinin , and A. Moschetta , “Visceral Adiposity and Cancer: Role in Pathogenesis and Prognosis,” Nutrients 13 (2021): 2101. 34205356 10.3390/nu13062101 PMC8234141

[48] P. Babaei and R. Hoseini , “Exercise Training Modulates Adipokine Dysregulations in Metabolic Syndrome,” Sports Medicine and Health Science 4 (2022): 18–28. 35782776 10.1016/j.smhs.2022.01.001 PMC9219261

[49] L. J. Mailing , J. M. Allen , T. W. Buford , C. J. Fields , and J. A. Woods , “Exercise and the Gut Microbiome: A Review of the Evidence, Potential Mechanisms, and Implications for Human Health,” Exercise and Sport Sciences Reviews 47 (2019): 75–85. 30883471 10.1249/JES.0000000000000183

[50] K. Tanha , A. Mottaghi , M. Nojomi , et al., “Investigation on Factors Associated With Ovarian Cancer: An Umbrella Review of Systematic Review and Meta‐Analyses,” Journal of Ovarian Research 14 (2021): 153. 34758846 10.1186/s13048-021-00911-z PMC8582179

[51] A. Giannini , I. Cuccu , T. G. D'Auge , et al., “The Great Debate: Surgical Outcomes of Laparoscopic Versus Laparotomic Myomectomy. A Meta‐Analysis to Critically Evaluate Current Evidence and Look Over the Horizon,” European Journal of Obstetrics & Gynecology and Reproductive Biology 297 (2024): 50–58. 38581885 10.1016/j.ejogrb.2024.03.045

[52] A. Giannini , T. Golia D'Augè , G. Bogani , et al., “Uterine Sarcomas: A Critical Review of the Literature,” European Journal of Obstetrics & Gynecology and Reproductive Biology 287 (2023): 166–170. 37348383 10.1016/j.ejogrb.2023.06.016

[53] S. Vafaei , S. Alkhrait , Q. Yang , M. Ali , and A. Al‐Hendy , “Empowering Strategies for Lifestyle Interventions, Diet Modifications, and Environmental Practices for Uterine Fibroid Prevention; Unveiling the LIFE UP Awareness,” Nutrients 16, no. 6 (2024): 807. 38542717 10.3390/nu16060807 PMC10975324

