# Supplementary material for: The Association Between Physical Activity and Uterine Leiomyoma and Its Symptoms: A Systematic Review and Meta‐Analysis
Source: Health Sci Rep. 2025 Feb 23;8(2):e70487. doi: 10.1002/hsr2.70487 (PMC11847712; doi:10.1002/hsr2.70487)
Supplement: Supplementary file 1 — Supporting information. [file HSR2-8-e70487-s001.docx]

**The association between physical activity and uterine leiomyoma and its symptoms: a systematic review and meta-analysis**

**Supplementary Material**

**Table S1.** PRISMA Checklist of items to include when reporting a systematic review or meta-analysis.

| Section/Topic | # | Checklist item | Reported on page # |
| --- | --- | --- | --- |
| TITLE | | | |
| Title | 1 | Identify the report as a systematic review, meta-analysis, or both. | p.1 |
| ABSTRACT | | | |
| Structured summary | 2 | Provide a structured summary including, as applicable: background; objectives; data sources; study eligibility criteria, participants, and interventions; study appraisal and synthesis methods; results; limitations; conclusions and implications of key findings; systematic review registration number. | p.3 |
| INTRODUCTION | | | |
| Rationale | 3 | Describe the rationale for the review in the context of what is already known. | p.5 |
| Objectives | 4 | Provide an explicit statement of questions being addressed with reference to participants, interventions, comparisons, outcomes, and study design (PICOS). | p.6 |
| METHODS | | | |
| Protocol and registration | 5 | Indicate if a review protocol exists, if and where it can be accessed (e.g., Web address), and, if available, provide registration information including registration number. | p.6 |
| Eligibility criteria | 6 | Specify study characteristics (e.g., PICOS, length of follow-up) and report characteristics (e.g., years considered, language, publication status) used as criteria for eligibility, giving rationale. | p.6 |
| Information sources | 7 | Describe all information sources (e.g., databases with dates of coverage, contact with study authors to identify additional studies) in the search and date last searched. | p.6 |
| Search | 8 | Present full electronic search strategy for at least one database, including any limits used, such that it could be repeated. | p.6 |
| Study selection | 9 | State the process for selecting studies (i.e., screening, eligibility, included in systematic review, and, if applicable, included in the meta-analysis). | p.7 |
| Data collection process | 10 | Describe method of data extraction from reports (e.g., piloted forms, independently, in duplicate) and any processes for obtaining and confirming data from investigators. | p.7 |
| Data items | 11 | List and define all variables for which data were sought (e.g., PICOS, funding sources) and any assumptions and simplifications made. | p.7 |
| Risk of bias in individual studies | 12 | Describe methods used for assessing risk of bias of individual studies (including specification of whether this was done at the study or outcome level), and how this information is to be used in any data synthesis. | p.7 |
| Summary measures | 13 | State the principal summary measures (e.g., risk ratio, difference in means). | p.7 |
| Synthesis of results | 14 | Describe the methods of handling data and combining results of studies, if done, including measures of consistency (e.g., I^2^) for each meta-analysis. | p.7 |
| Risk of bias across studies | 15 | Specify any assessment of risk of bias that may affect the cumulative evidence (e.g., publication bias, selective reporting within studies). | p.7 |
| Additional analyses | 16 | Describe methods of additional analyses (e.g., sensitivity or subgroup analyses, meta-regression), if done, indicating which were pre-specified. | p.8 |
| RESULTS | | | |
| Study selection | 17 | Give numbers of studies screened, assessed for eligibility, and included in the review, with reasons for exclusions at each stage, ideally with a flow diagram. | p.8 |
| Study characteristics | 18 | For each study, present characteristics for which data were extracted (e.g., study size, PICOS, follow-up period) and provide the citations. | p.8 and p.9 |
| Risk of bias within studies | 19 | Present data on risk of bias of each study and, if available, any outcome-level assessment (see Item 12). | p.9 |
| Results of individual studies | 20 | For all outcomes considered (benefits or harms), present, for each study: (a) simple summary data for each intervention group and (b) effect estimates and confidence intervals, ideally with a forest plot. | p.9 |
| Synthesis of results | 21 | Present the main results of the review. If meta-analyses are done, include for each, confidence intervals and measures of consistency. | p.9 |
| Risk of bias across studies | 22 | Present results of any assessment of risk of bias across studies (see Item 15). | p.9 |
| Additional analysis | 23 | Give results of additional analyses, if done (e.g., sensitivity or subgroup analyses, meta-regression [see Item 16]). | p.9 and p.10 |
| DISCUSSION | | | |
| Summary of evidence | 24 | Summarize the main findings including the strength of evidence for each main outcome; consider their relevance to key groups (e.g., health care providers, users, and policy makers). | p.10 |
| Limitations | 25 | Discuss limitations at study and outcome level (e.g., risk of bias), and at review level (e.g., incomplete retrieval of identified research, reporting bias). | p.12 |
| Conclusion | 26 | Provide a general interpretation of the results in the context of other evidence, and implications for future research. | p.13 |
| FUNDING | | | |
| Funding | 27 | Describe sources of funding for the systematic review and other support (e.g., supply of data); role of funders for the systematic review. | NA |

NA: Not applicable.

**Table S2.** Search strategy used in each database.

| Search updated at 01/10/2025 |
| --- |
| **MEDLINE-Pubmed (n=2052)** |
| (leiomyoma* OR "uterine fibroids" OR fibromyoma OR "uterine fibroma") AND (exercise OR "physical activity" OR sedentar* OR fitness OR athlet* OR movement OR inactiv* OR MVPA OR LTPA OR HIIT OR aerobic OR training OR runn* OR swim* OR yoga OR taichi OR tai-chi OR "tai chi" OR walking OR sport* OR BMI OR “body mass” OR “body fat” OR obes* OR overweight* OR “excess weight” OR “lean mass” OR “fat mass” OR “body composition” OR “waist circumference” OR adiposity OR fatness) |
| **EMBASE-Scopus (n=2229)** |
| TITLE-ABS-KEY (leiomyoma*  OR  "uterine fibroids"  OR  fibromyoma  OR  "uterine fibroma")  AND  TITLE-ABS-KEY (exercise  OR  "physical activity"  OR  sedentar*  OR  fitness  OR  athlet*  OR  movement  OR  inactiv*  OR  mvpa  OR  ltpa  OR  hiit  OR  aerobic  OR  training  OR  runn*  OR  swim*  OR  yoga  OR  taichi  OR  tai-chi  OR  "tai chi"  OR  walking  OR  sport* OR BMI OR “body mass” OR “body fat” OR obes* OR overweight* OR “excess weight” OR “lean mass” OR “fat mass” OR “body composition” OR “waist circumference” OR adiposity OR fatness) |
| **Web of Science (n=4489)** |
| TS=(leiomyoma*  OR "uterine fibroids"  OR fibromyoma  OR "uterine fibroma")  AND TS=(exercise  OR "physical activity"  OR sedentar*  OR fitness  OR athlet*  OR movement  OR inactiv*  OR MVPA  OR LTPA  OR HIIT  OR aerobic  OR training  OR runn*  OR swim*  OR yoga  OR taichi  OR tai-chi  OR "tai chi"  OR walking  OR sport* OR BMI OR “body mass” OR “body fat” OR obes* OR overweight* OR “excess weight” OR “lean mass” OR “fat mass” OR “body composition” OR “waist circumference” OR adiposity OR fatness) |
| **Scielo.org (n=425)** |
| (leiomyoma) OR (fibromyoma) OR (uterine fibroids) OR (uterine fibroma) OR (myoma) |
| **Cochrane Library (n=248)** |
| (leiomyoma* OR "uterine fibroids" OR fibromyoma OR "uterine fibroma" ) AND (exercise OR "physical activity" OR sedentar* OR fitness OR athlet* OR movement OR inactiv* OR mvpa OR ltpa OR hiit OR aerobic OR training OR runn* OR swim* OR yoga OR taichi OR tai-chi OR "tai chi" OR walking OR sport* OR BMI OR “body mass” OR “body fat” OR obes* OR overweight* OR “excess weight” OR “lean mass” OR “fat mass” OR “body composition” OR “waist circumference” OR adiposity OR fatness) |
|  |
| **Total without duplicates**: 5730 |

**Table S3.** Adapted Newcastle‒Ottawa Scale for Cross-Sectional Studies.

| **Cluster** | **Question** | **Possible answers** |
| --- | --- | --- |
| **Selection (Maximum of 4 stars)** | 1) Representativeness of the sample | **a) representative of the average in the target population. * (all subjects or random sampling)** |
|  |  | **b) somewhat representative of the average in the target population. * (nonrandom sampling)** |
|  |  | c) selected group of users e.g., those with diabetes or other disorders only, age limit, obese BMI only etc. *Where inclusion/exclusion criteria are restrictive* |
|  |  | d) no description of the sampling strategy. |
|  | 2) Sample size: | **a) justified and satisfactory. *** |
|  |  | b) not justified |
|  | 3) Nonrespondents: | **a) comparability between respondents and nonrespondents characteristics is established, and the response rate is satisfactory. *** |
|  |  | b) the response rate is unsatisfactory, or the comparability between respondents and nonrespondents is unsatisfactory |
|  |  | c) no description of the response rate or the characteristics of the responders and the nonresponders |
|  | 4) Ascertainment of the exposure (risk factor): | **a) validated measurement tool. *** |
|  |  | **b) nonvalidated measurement tool, but the tool is available or described.*** |
|  |  | c) no description of the measurement tool. |
| **Comparability (Maximum of 2 stars)** | 5) The subjects in different outcome groups are comparable, based on the study design or analysis. Confounding factors are controlled. | **a) study controls for age*** |
|  |  | **b) study controls for any additional factor*** |
|  |  | c) no factors controlled for |
| **Outcome (Maximum of 2 stars)** | 6) Assessment of outcome | **a) independent blind assessment (e.g., explicitly measured for the purposes of the research)*** |
|  |  | **b) record linkage (e.g., routine medical records)*** |
|  |  | c) **self-report*** |
|  |  | d) no description |
|  | 7) Statistical test | **a) the statistical test used to analyze the data are clearly described and appropriate, and the measurement of the association is presented, including confidence intervals and the probability level (p value). *** |
|  |  | b) the statistical test is not appropriate, not described or incomplete. |

**Table S4.** Excluded studies by reason for exclusion (n = 95).

| **No data of interest (n = 75)** |
| --- |
| 1. AbdElmagied, A.M., et al. Am J Obstet Gynecol, 2016. doi**:**10.1016/j.ajog.2016.04.001 |
| 2. Ahrendt, H.J., et al. Arch Gynecol Obstet, 2016. doi**:**10.1007/s00404-015-3930-8 |
| 3. Atalyan, A.V., et al. Int J Biomed. 2021. doi**:**10.21103/Article11(4)_OA18 |
| 4. Babaeva, N.I., et al. Acta Biomedica Scientifica, 2024. doi**:**10.29413/ABS.2024-9.4.4 |
| 5. Barbosa, L.F.d.S. Doctoral Thesis. Universidade da Beira Interior, Covilhã, Portugal. 2012. |
| 6. Boclin, K.L.S., et al. Rev Saude Publica, 2015. doi**:**10.1590/s0034-8910.2015049005898 |
| 7. Borah, B.J., et al. Am J Obstet Gynecol. 2013. doi: 10.1016/j.ajog.2013.07.017 |
| 8. Bray, M.J., et al. Maturitas, 2018. doi**:**10.1016/j.maturitas.2018.05.003 |
| 9. Brewster, L.M., et al. Cureus, 2022. doi**:**10.7759/cureus.30740 |
| 10. Callegari, L.S., et al. Womens Health Issues, 2019. doi**:**10.1016/j.whi.2018.08.005 |
| 11. Chen, C.R., et al. Am J Epidemiol, 2001. doi**:**10.1093/aje/153.1.20 |
| 12. Dai, Y., et al. Chin Med J (Engl), 2024. doi**:**10.1097/cm9.0000000000002971 |
| 13. Dubinskaya, E.D., et al. Akush Ginekol (Mosk) 2024. doi**:**10.18565/aig.2024.128 |
| 14. Faerstein, E., et al. Am J Epidemiol, 2001. doi**:**10.1093/aje/153.1.1 |
| 15. Fatima, W., et al. Womens Health Reports, 2024. doi**:**10.1089/whr.2023.0145 |
| 16. Fuldeore, M.J., et al. Int J Womens Health, 2017. doi**:**10.2147/ijwh.S133212 |
| 17. Giri, A., et al. Reproductive Sciences, 2017 |
| 18. Gulersen, M., et al. Am J Obstet Gynecol MFM, 2024. doi**:**10.1016/j.ajogmf.2024.101415 |
| 19. Güzel, A.İ., et al. J Obstet Gynaecol Res, 2015. doi**:**10.1111/jog.12635 |
| 20. Haan, Y.C., et al. Journal of Clinical Hypertension, 2018. doi**:**10.1111/jch.13253 |
| 21. Haan, Y.C., et al. American Journal of Hypertension, 2015. doi**:**10.1093/ajh/hpu183 |
| 22. Han, X., et al. BMC Womens Health, 2024. doi**:**10.1186/s12905-024-03196-8 |
| 23. Hanfy, H.M., et al. Fizjoterapia Polska, 2021 |
| 24. Harmon, Q.E., et al. J Clin Endocrinol Metab, 2024. doi**:**10.1210/clinem/dgae036 |
| 25. Hong, D.G., et al. Korean journal of fertility and sterility, 2006 |
| 26. Huseman-Plascencia, L.A., et al. J Endometr Pelvic Pain Disord, 2021. doi**:**10.1177/22840265211065226 |
| 27. Ilaria, S., et al. Curr Med Res Opin, 2018. doi**:**10.1080/03007995.2018.1505606 |
| 28. Impis Oglou, M., et al. Am J Obstet Gynecol MFM, 2024. doi**:**10.1016/j.ajogmf.2024.101452 |
| 29. Khamaiseh, S., et al. Am J Obstet Gynecol., 2025. doi**:**10.1016/j.ajog.2024.06.051 |
| 30. Kim, M.J., et al. PLoS ONE, 2024. doi**:**10.1371/journal.pone.0291157 |
| 31. Koehler, G., et al. Archives of Gynecology and Obstetrics, 2019. doi**:**10.1007/s00404-019-05344-0 |
| 32. Korkmaz, V., et al. Irish Journal of Medical Science, 2016. doi**:**10.1007/s11845-015-1343-0 |
| 33. Laberge, P.Y., et al. Current Medical Research and Opinion, 2016. doi**:**10.1185/03007995.2015.1107534 |
| 34. Lambertino, A., et al. Environ Res, 2011. doi**:**10.1016/j.envres.2011.01.006 |
| 35. Langton, C.R., et al. JAMA Network Open, 2024. doi**:**10.1001/jamanetworkopen.2024.4185 |
| 36. Lee, J.-E., et al. Current Medical Research and Opinion, 2018. doi**:**10.1080/03007995.2018.1462783 |
| 37. Li, B., et al. Front Cardiovasc Med, 2022. doi**:**10.3389/fcvm.2022.975920 |
| 38. Liu, X., et al. Front Physiol, 2023. doi**:**10.3389/fphys.2023.1197658 |
| 39. Luoto, R., et al. Maturitas, 2000. doi**:**10.1016/s0378-5122(00)00160-2 |
| 40. Ming, W.-K., et al. Health and Quality of Life Outcomes, 2019. doi**:**10.1186/s12955-019-1153-6 |
| 41. Mitro, S.D., et al. Fertil Steril, 2022. doi**:**10.1016/j.fertnstert.2022.06.028 |
| 42. Moore, A.B., et al. J Reprod Med, 2008 |
| 43. Ndebele, S., et al. Int J Environ Res Public Health., 2024. doi**:**10.3390/ijerph21020222 |
| 44. Okesola, M.A., et al. *The Multi-risk Factors Promoting Uterine Fibroids in Women*. *SEB-SDG 2023*. |
| 45. Olusi, A.M., et al. Nigerian Health Journal, 2024. doi**:**10.60787/tnhj-24-1-765 |
| 46. Parazzini, F. Maturitas, 2006. doi**:**10.1016/j.maturitas.2006.01.013 |
| 47. Parazzini, F., et al. Eur J Epidemiol, 2004. doi**:**10.1023/b:ejep.0000020448.43323.2a |
| 48. Parazzini, F., et al. Obstet Gynecol, 1988. doi**:**10.1097/00006250-198812000-00008 |
| 49. Park, S.A., et al. Heliyon, 2024. doi**:**10.1016/j.heliyon.2023.e23759 |
| 50. Peddada, S.D., et al. Proc Natl Acad Sci U S A., 2008. doi**:**10.1073/pnas.0808188105 |
| 51. Qu, Y., et al. Front Endocrinol (Lausanne), 2023. doi**:**10.3389/fendo.2023.1133260 |
| 52. Rubio, E.M., et al. J Womens Health (Larchmt), 2023. doi**:**10.1089/jwh.2022.0094 |
| 53. Sadlonova, J., et al. Int J Gynaecol Obstet, 2008. doi**:**10.1016/j.ijgo.2008.01.022 |
| 54. Samadi, A.R., et al. American Journal of Public Health, 1996. doi**:**10.2105/AJPH.86.6.858 |
| 55. Sarkodie, B.D., et al. Fertil Res Pract, 2016. doi**:**10.1186/s40738-016-0022-9 |
| 56. Sharami, S.H., et al. Pakistan Journal of Medical and Health Sciences, 2020 |
| 57. Sharami, S.H., et al. Arch Iran Med, 2019 |
| 58. Silva, R.O.d., et al. Revista Brasileira de Ginecologia e Obstetrícia, 2016. doi**:**10.1055/s-0036-1593833 |
| 59. Smailova, L.K., et al. Systematic Reviews in Pharmacy, 2020. doi**:**10.5530/srp.2020.2.27 |
| 60. Song, S., et al. BMC Women's Health, 2023. doi**:**10.1186/s12905-023-02447-4 |
| 61. Spies, J.B., et al. Obstetrics and Gynecology, 2010. doi**:**10.1097/AOG.0b013e3181ed36b3 |
| 62. Stewart, E.A., et al. J Womens Health (Larchmt), 2013. doi**:**10.1089/jwh.2013.4334 |
| 63. Subramaniyam, N.K., et al. Journal of Young Pharmacists, 2020. doi**:**10.5530/jyp.2020.12.17 |
| 64. Sun, Y., et al. BMJ Open, 2023. doi**:**10.1136/bmjopen-2023-073592 |
| 65. Tatlici, T.K., et al. Rev Assoc Med Bras (1992), 2024. doi**:**10.1590/1806-9282.20231359 |
| 66. Templeman, C., et al. Fertil Steril, 2009. doi**:**10.1016/j.fertnstert.2008.08.074 |
| 67. Wilson, L.F., et al. Human Reproduction, 2024. doi**:**10.1093/humrep/deae162 |
| 68. Wise, L.A., et al. American Journal of Epidemiology, 2004. doi**:**10.1093/aje/kwh016 |
| 69. Wise, L.A., et al. American Journal of Obstetrics and Gynecology, 2013. doi**:**10.1016/j.ajog.2012.12.034 |
| 70. Wu, Q., et al. Clinical Imaging, 2024. doi**:**10.1016/j.clinimag.2023.110028 |
| 71. Xu, H., et al. Frontiers in Endocrinology, 2024. doi**:**10.3389/fendo.2024.1373724 |
| 72. Yang, Y., et al. J Womens Health (Larchmt), 2014. doi**:**10.1089/jwh.2013.4690 |
| 73. Zhang, L., et al. BMJ Open, 2025. doi**:**10.1136/bmjopen-2024-085671 |
| 74. Zhou, M., et al. Asia Pac J Clin Nutr, 2020. doi**:**10.6133/apjcn.202012_29(4).0012 |
| 75. 전상식*.* Clinical and Experimental Reproductive Medicine, 2006 |

| **Reviews (n = 18)** |
| --- |
| 1. Aninye, I.O., et al. J Womens Health (Larchmt), 2021. doi:10.1089/jwh.2021.0280 |
| 2. Flake, G.P., et al. Environ Health Perspect, 2003. doi:10.1289/ehp.5787 |
| 3. Keizer, A.L., et al. J Obstet Gynaecol, 2024. doi:10.1080/01443615.2023.2288225 |
| 4. Morhason-Bello, I.O., et al. BMJ Open, 2022. doi:10.1136/bmjopen-2021-052053 |
| 5. Pavone, D., et al. Best Pract Res Clin Obstet Gynaecol, 2018. doi:10.1016/j.bpobgyn.2017.09.004 |
| 6. Ponomarenko, M.S., et al. Akush Ginekol (Mosk), 2024. doi:10.18565/aig.2023.275 |
| 7. Qin, H., et al. J Epidemiol Community Health, 2021. doi:10.1136/jech-2019-213364 |
| 8. Rongières, C. J Gynecol Obstet Biol Reprod (Paris), 1999 |
| 9. Salehi, A.M., et al. J Gynecol Obstet Hum Reprod, 2023. doi:10.1016/j.jogoh.2022.102517 |
| 10. Schwartz, S.M., et al. Environ Health Perspect, 2000. doi:10.1289/ehp.00108s5821 |
| 11. Sefah, N., et al. Frontiers in Pharmacology, 2023. doi:10.3389/fphar.2022.1045783 |
| 12. Sparic, R., et al. Int J Fertil Steril, 2016. doi:10.22074/ijfs.2015.4599 |
| 13. Stewart, E.A., et al. Bjog, 2017. doi:10.1111/1471-0528.14640 |
| 14. Vafaei, S., et al. Nutrients, 2024. doi:10.3390/nu16060807 |
| 15. Vafaei, S., et al. Int J Mol Sci, 2023. doi:10.3390/ijms242115972 |
| 16. Vilos, G.A., et al. J Obstet Gynaecol Can, 2015. doi:10.1016/S1701-2163(15)30338-8 |
| 17. Vollenhoven, B.J., et al. Br J Obstet Gynaecol, 1990. doi:10.1111/j.1471-0528.1990.tb01804.x |
| 18. Wise, L.A., et al. Clin Obstet Gynecol, 2016. doi:10.1097/grf.0000000000000164 |

| **Duplicated data (n = 2)** |
| --- |
| 1. Dragomir, A.D., et al. Reprod Sci, 2010. doi**:**10.1177/1933719110376979 |
| 2. Sung, J.-H., et al. Diabetes Metab J, 2024. doi**:**10.4093/dmj.2023.0444 |

**Table S5.** Quality assessment scores for cross-sectional, cohort and case‒control studies.

| Studies | Newcastle Ottawa Scale for **cross-sectional** **studies** question number and score allocated | | | | | | | | | Total Stars (max. 8) |
| --- | --- | --- | --- | --- | --- | --- | --- | --- | --- | --- |
|  | 1 | 2 | 3 | 4 | 5 | | 6 | 7 | |  |
| Baird et al. 2007 | a* | a* | a* | a* | a*+b* | | a* | a* | | 8 |
| Borah et al. 2013 | a* | a* | b | a* | c | | c* | a* | | 5 |
| Wyshak et al. 1986 | a* | a* | c | b* | a*+b* | | c* | a* | | 7 |
| Studies | Newcastle Ottawa Scale for **cohort** **studies** question number and score allocated | | | | | | | | | Total Stars (max. 9) |
|  | 1 | 2 | 3 | 4 | 5 | 6 | | 7 | 8 |  |
| Huang et al. 2017 | c | a* | b* | a* | c | b* | | c | a* | 5 |
| Jacoby et al. 2014 | c | a* | b* | a* | a*+b* | a* | | a* | b* | 8 |
| Kim et al. 2023 | a* | a* | c | b | c | b* | | b | a* | 4 |
| Kim et al. 2024 | c | a* | b* | a* | c | b* | | b | b* | 5 |
| Uimari et al., 2016 | a* | a* | c | a* | c | c | | a* | c | 4 |
| Wise et al., 2016 | a* | a* | b* | a* | c | c | | a* | d | 5 |
| Studies | Newcastle Ottawa Scale for **case‒control studies** question number and score allocated | | | | | | | | | Total Stars (max. 9) |
|  | 1 | 2 | 3 | 4 | 5 | 6 | | 7 | 8 |  |
| Abdel Aziz et al., 2016 | a* | a* | a* | a* | c | b* | | a* | c | 6 |
| Feofilova et al., 2018 | a* | a* | b | a* | c | c | | a* | c | 4 |
| He et al. 2013 | a* | a* | a* | a* | a*+ b* | b* | | a* | c | 8 |
| Muawad et al., 2022 | a* | a* | a* | a* | a*+b* | c | | a* | c | 7 |
| Shen et al., 2013 | a* | a* | a* | a* | c | c | | a* | c | 5 |
| Tak et al., 2016 | a* | a* | a* | a* | c | c | | a* | c | 5 |

**Table S6.** Grades of Recommendations, Assessment, Development, and Evaluation.

| **Study** | **Certainty in the evidence^a^** | **Comments** |
| --- | --- | --- |
| Abdel Aziz et al., 2016 | ØOOO  VERY LOW  Due to imprecision^b^ and risk of bias | Case‒control study; observational analysis, small sample size, uncontrolled confounding |
| Baird et al., 2007 | ØØØO  MODERATE | Cross-sectional study; observational analysis, dose‒response gradient, controlled confounding |
| Borah et al., 2013 | ØOOO  VERY LOW  Due to risk of bias | Cross-sectional study; observational analysis, uncontrolled confounding |
| Feofilova et al., 2018 | ØOOO  VERY LOW  Due to imprecision^b^ and risk of bias | Case‒control study; observational analysis, small sample size, uncontrolled confounding |
| He et al., 2013 | ØOOO  VERY LOW  Due to imprecision^b^ | Case‒control study; observational analysis, controlled confounding but small sample size |
| Huang et al., 2017 | ØOOO  VERY LOW  Due to imprecision^b^ and risk of bias | Cohort study; observational analysis, small sample size, uncontrolled confounding |
| Jacoby et al., 2014 | ØØOO  LOW | Cohort study; observational analysis, controlled confounding |
| Kim et al., 2023 | ØOOO  VERY LOW  Due to risk of bias | Cohort study; observational analysis, uncontrolled confounding |
| Kim et al., 2024 | ØOOO  VERY LOW  Due to risk of bias | Cohort study; observational analysis, uncontrolled confounding |
| Muawad et al., 2022 | ØØOO  LOW | Case‒control study; observational analysis, controlled confounding |
| Shen et al., 2013 | ØOOO  VERY LOW  Due to risk of bias | Case‒control study; observational analysis, uncontrolled confounding |
| Tak et al., 2016 | ØOOO  VERY LOW  Due to risk of bias | Case‒control study; observational analysis, uncontrolled confounding |
| Uimari et al., 2016 | ØOOO  VERY LOW  Due to risk of bias | Cohort study; observational analysis, uncontrolled confounding |
| Wise et al., 2016 | ØOOO  VERY LOW  Due to risk of bias | Cohort study; observational analysis, uncontrolled confounding |
| Wyshak et al., 1986 | ØØOO  LOW | Cross-sectional study; observational analysis, controlled confounding |

^a^ GRADE level for certainty of evidence: ‘high’ indicates that we are very confident that the true effect lies close to that of the estimate of the effect; ‘moderate’ indicates that we are moderately confident in the effect estimate (the true effect is likely to be close to the estimate of the effect, but there is a possibility that it is substantially different); ‘low’ indicates that our confidence in the effect estimate is limited (the true effect may be substantially different from the estimate of the effect); and ‘very low’ indicates that we have very little confidence in the effect estimate (the true effect is likely to be substantially different from the estimate of effect). ^b^ Rated down one level due to imprecision, as the sample size is less than the optimal information size of 400 individuals.

**Table S7.** Sensitivity analysis of the meta-analysis of the frequency of physical activity and UL.

-----------------------------------------------------------------------

Study omitted | Estimate [95% Conf. Interval]

-----------------------------------------------------------------------

Feofilova et al., 2018 | .96100760 .85713482 1.0774683

He et al., 2013 | .91643035 .80688614 1.0408465

Kim et al., 2023 | .89744717 .77144206 1.0440335

Muawad et al., 2022 | .92913377 .80872011 1.0674764

Shen et al., 2013 | .90933621 .78108943 1.0586398

Tak et al., 2016 | .93560636 .81231034 1.0776168

Uimari et al., 2016 | .90112650 .78965205 1.0283377

Wyshak et al., 1986 | .97625762 .87999797 1.0830468

-----------------------------------------------------------------------

Combined | .92955241 .82261547 1.0503907

-----------------------------------------------------------------------

**Table S8.** Sensitivity analysis of the meta-analysis of the intensity of physical activity and UL.

---------------------------------------------------------------------

Study omitted | Estimate [95% Conf. Interval]

---------------------------------------------------------------------

**Intermediate vs. Low PA**

Abdel Aziz et al., 2016 | .94075227 .86361587 1.0247782

Baird et al., 2007 (afro) | .86940074 .76731986 .9850620

Baird et al., 2007 (cauc) | .87395948 .78163028 .9771950

He et al., 2013 | .88409674 .79565150 .9823736

Uimari et al., 2016 | .86417210 .77227563 .9670037

**High vs. Low PA**

Abdel Aziz et al., 2016 | .93078691 .85481709 1.0135083

Baird et al., 2007 (afro) | .88359892 .78377420 .9961376

Baird et al., 2007 (cauc) | .89989150 .80737603 1.0030081

He et al., 2013 | .88422292 .79589295 .9823558

Uimari et al., 2016 | .87643760 .78022981 .9845085

Wise et al., 2016 | .86399931 .75362319 .9905411

---------------------------------------------------------------------

Combined | .88993618 .80252048 .9868737

---------------------------------------------------------------------
